# Condom Use and its Associated Factors Among Iranian Youth: Results From a Population-Based Study

**DOI:** 10.15171/ijhpm.2018.65

**Published:** 2018-08-04

**Authors:** Samira Hosseini Hooshyar, Mohammad Karamouzian, Ali Mirzazadeh, Ali Akbar Haghdoost, Hamid Sharifi, Mostafa Shokoohi

**Affiliations:** ^1^HIV/STI Surveillance Research Center, and WHO Collaborating Center for HIV Surveillance, Institute for Futures Studies in Health, Kerman University of Medical Sciences, Kerman, Iran.; ^2^The Kirby Institute, UNSW Sydney, Sydney, NSW, Australia.; ^3^School of Population and Public Health, Faculty of Medicine, University of British Columbia, Vancouver, BC, Canada.; ^4^Department of Epidemiology and Biostatistics, University of California San Francisco, San Francisco, CA, USA.; ^5^Department of Epidemiology & Biostatistics, Schulich School of Medicine & Dentistry, The University of Western Ontario, London, ON, Canada.

**Keywords:** Condom, Sexual Behaviour, Educational Programs, Young Adult, Iran

## Abstract

**Background:** Given the young structure of Iran’s population and the fact that extramarital sexual relationships are both prohibited by legislation and shunned by society and religion, examining condom use practices among Iranian youth is highly important. The aim of this study was to explore condom use and its correlates among Iranian young adults.

**Methods:** In a sample of 3,045 individuals aged 19-29 who were recruited from a nation-wide study, we analyzed data from 633 participants who reported a history of extramarital sex. Subjects were asked about their condom use practices during their last penetrative sex. Data were collected through a self-administered questionnaire where the respondents completed the survey on their own and passed it to trained gender-matched interviewers. Multivariable regression models were constructed to report adjusted odds ratios (AOR) along with 95% CI.

**Results:** Of the 633 participants, 222 (35.1%) reported condom use at last sex. Men reported significantly higher condom use than women (38.5% vs. 25.7%). Having a stable job (AOR = 1.86, 95% CI: 1.01, 3.43), higher knowledge of condom use (AOR = 1.57, 95% CI: 1.03, 2.37) and sexual transmission of HIV (AOR = 1.83, 95% CI: 1.18, 2.85) were positively associated with condom use at last sex. Conversely, experience of sex under the influence of substances (AOR = 0.66, 95% CI: 0.45, 0.94) was significantly associated with reduced odds of condom use at last sex.

**Conclusion:** This study shows that only one out of every three young adults reported using condoms at last sex. While educational programs are helpful, multi-sectoral approaches (eg, individual-, community-, and structural-level interventions) are required to change sexual behaviours towards safe sex practices and reinforce negotiating condom use among youth.

## Background

Young adulthood is commonly described as a period of new experiences which might include risky behaviours.^1,2^ These behaviours could be one or a combination of condomless sex, alcohol consumption or drug use before sex and having multiple sexual partners.^3,4^ Engaging in such behaviours raises concerns about the spread of sexually transmitted infections (STIs) as well as HIV/AIDS among this population.^2,5^

Of the approximately 80 million people that reside in Iran, roughly 25.1% are 15-29 years of age.^6^ The mean age at first marriage has now reached the late 20s for both men and women in Iran^7,8^; a phenomena that could encourage engaging in extramarital sex among numerous young adults.^7^ Additionally, new technologies like internet services have extended people’s communications beyond the traditional context of Iran which might enhance opportunities for sexual exploration among unmarried young people.^3^ This is particularly concerning given the fact that the pattern of HIV transmission in Iran has been recently shifting from intravenous drug use to high risk sexual practices.^9,10^

Within this context, sexual health education is an area in which investing and program planning is vital.^11^ A recent study among a random sample of 15 to 45-year-old men and women found that young and unmarried individuals benefit from culturally-adapted education on puberty, the reproductive system, and STIs/HIV/AIDS right before their first sexual experiences.^12^

Despite the known benefit of youth-specific sexual health education, it is often a controversial and neglected topic in Iran.^13^ Conservative cultures and stringent traditions have posed a significant challenge to open discourse and research on sexual education for several decades. However, despite these challenges, researchers are still investigating this issue because of concerns about the increased prevalence of condomless sex and its associated consequences among young adults.^14^

Behavioural interventions have been widely used elsewhere to reduce risk for HIV infection. Condom use promotion is a commonly used interventional strategy that has been proven to effectively diminish high-risk behaviours.^15,16^ However, condom use behaviours and determinants have been shown to be complex and dependent on several individual, socio-cultural and environmental factors.^17-19^ For example, studies in sub-Saharan Africa suggest that despite varying condom use patterns across different socio-demographic groups, some factors such as perceived or high self-efficacy to use condoms, safe sex negotiation skills, being men, and condom accessibility could predict condom use among young people.^1,20^

There is a narrow body of evidence about condom use and its correlates among young adults in Iran. The existing body of literature on young people’s sexual behaviour in Iran is limited to certain subgroups (eg, Women at risk for HIV),^21-23^ or certain urban settings (eg, Tehran) or specific gender sub-groups (eg, young boys)^24,25^; findings that may not be generalizable to the young adult population of the country. Therefore, this study aimed to determine the prevalence and correlates of condom use among this population using a nation-wide sample of young adults.

## Methods

### Study Population and Sampling

A cross-sectional survey was carried out between January and March 2013 in 13 provinces in Iran in order to assess the HIV-related knowledge, attitudes, and practices among young population in three age categories (15-18, 19-24 and 25-29 years old). A total of 4950 participants were recruited through a non-random multistage sampling approach. As the survey did not aim to explore condom use outcomes among subjects aged 15-18 years, questions on HIV-related risk practices were only asked of those aged 19 or older (n = 3045). Out of these participants, 633 young adults reported ever having extramarital sex identified by a single question: “Have you ever had extramarital sex?” Extramarital sex was defined as having “sex” outside the context of marriage whether the participant was or was not married.

The details of the study method and sampling strategies are published elsewhere.^11,26,27^ In brief, considering the geographic distribution of the provinces in the country, 13 provinces, including Iran’s capital Tehran, and 12 other provinces were selected for data collection. A total of 990 individuals in Tehran and 330 individuals in each of the 12 provinces were recruited. In each province, 70% of the participants were selected from the capital city and 30% from non-capital cities. In each city, 70% and 30% of the individuals were respectively recruited from urban and suburban areas. Empirical studies in Iran have shown that cultural and religious restrictions might limit reporting sensitive information such as sexual practices or intercourses, and that people are more likely to disclose this information in the street-based surveys in comparison with telephone or home-based surveys.^28,29^ As a result, participants were recruited from public places and crowded streets in urban areas. In fact, urban areas were divided in five regions (north, east, west, south and central) and two public places were selected from each region. In suburban areas, we determined three strata and one village was selected in each stratum. Interviews were conducted by trained, local and gender-matched interviewers in each location.

### Study Instrument

The development of the study instrument was informed by a review of the relevant literature as well as consultations with an advisory group of HIV experts and key informants at the Ministry of Health and Medical Education (MoHME), and was pilot-tested among 150 individuals in three provinces to check and ensure clarity, relevance, and accessibility. The questionnaire was a self-administered paper-and-pencil instrument that contained items about participants’ socio-demographic characteristics, condom and HIV-related knowledge, and lifetime sexual and high-risk sexual behaviours.

### Measures

#### Outcome Variable

To assess condom use at last sex, participants were asked a single question: “Did you use a condom the last time you had penetrative sex?” This variable was coded as “yes” if they reported using a condom at last sex; otherwise it was coded as “no.” Reasons for not using a condom were also asked from those who reported not using a condom.

#### Socio-demographic Variables

These variables included age groups (19-24, 25-29 years old); gender (men, women); the highest level of education (Illiterate [ie, could not read and write], less than high school diploma, undergraduate [AS/BS degrees], or graduate [ie, Masters degree and above]); occupation status (unemployed/housekeepers, employed in government sector or self-employed, laborer or other type of workers, students); and marital status (currently married, currently unmarried [ie, never married, separated, divorced and widowed]).

#### Sexual Behaviours Variables

We included the following two main variables in our analysis. I) the age at first sex was inquired about and coded as a binary variable (≤18 vs. >18 years old); II) whether or not the participant had ever had sex while high on drugs or alcohol. This was coded in a binary manner as “yes” when they reported having sex under such condition vs. “other options” (ie, no or cannot remember).

#### Condom-Related Knowledge

There were two statements related to condom use: (I) “condomless sex with an HIV-positive partner can transmit the HIV infection” and (II) “HIV can be prevented by consistent use of condoms during sex.” Both had three options: True, False, and I do not know. Along with reporting their individual frequencies, we created a single variable used for the analysis. Those who answered both questions correctly were considered as having correct knowledge related to condom effectiveness, while if they had at least one incorrect answer (either no or do not know answers) they were considered as having incorrect knowledge.

#### Knowledge of Sexual Transmission of HIV

Among questions on HIV-related knowledge, there was a question specifically assessing the knowledge of participants on the sexual route of HIV transmission: “Having penetrative sex with more than one partner/person increases the likelihood of HIV acquisition and transmission.” This statement had three options: yes, no and I do not know. Participants were categorized in yes group if they correctly answered the question. Also, if they incorrectly answered or did not know the answer, they were put into no and I do not know categories.

#### Statistical Analysis

Absolute and relative frequencies for categorical variables and mean and standard deviation for continuous variables were calculated. Condom use at last sex was reported for the overall sample and both men and women separately. Chi-square tests were used to compare the frequency of the outcome in sub-groups of the independent covariates of the study. Univariate logistic regression model was applied to estimate odds ratios (OR). Variables with a *P* value less than 0.20^30^ were entered into the multivariable regression model and adjusted odds ratios (AOR) along with 95% CI were reported. Statistical analyses were done using Stata v. 13 (StataCorp; College Station, TX, USA).

## Results

### Descriptive Statistics

In total, 633 (20.8%) reported extramarital sex and were included in this analysis. The mean age (SD) of the included participants was 24.4 (3.1); it was 24.5 (3.1) and 24.2 (3.0) for men and women respectively (*P* value = .376). More than three quarters (n = 488; 77.1%) were men and the rest were women. Most participants had a job in the government sector or ran their own job (n = 303; 48.0%), had more than a diploma (n = 230; 39.5% AS/BS degree and n = 277; 43.9% more than BS degree), and were currently unmarried (n = 447; 71.6%). A considerable proportion of the participants started having sex <19 years old (n = 170; 41.3%). Mean (SD) age at first sex for men and women was 19.0 (3.3) and 20.2 (3.2), respectively (*P* = .001). Of the whole sample, 40.0% reported having sex while high on drugs or alcohol. A large proportion of participants (n = 570; 90.6%) correctly knew that condomless sex with an HIV-positive partner could transmit HIV and around three-quarters knew that HIV could be prevented by using condom during sex. Overall, 70.7% had good knowledge about the efficacy of the condom. Around three-quarter of the participants knew that having sex with more than one person increases the likelihood of HIV infection (Table 1).

**Table 1:** The Prevalence of Condom Use at Last Sex Overall and Stratified by Gender Across Demographic and Behavioural Variables Among Young Adults (Analytic Sample = 633; Men = 488; Women = 145)

**Variables**	**N**	**Overall, No. (%)** ^a^	**Men, No. (%)**	**Women, No. (%)**
Age group				
19-24 years	318	101 (32.3)	82 (35.0)	16 (21.6)
25-29 years	314	121 (39.2)^e^	100 (41.2)	20 (30.8)
Educational attainment				
Illiterate	30	13 (43.3)	9 (50.0)	4 (33.3)
Diploma and less	94	33 (36.3)	26 (34.2)	7 (46.7)
Undergraduate	230	78 (34.5)	66 (35.3)	10 (28.6)
Graduate	277	98 (35.8)	81 (41.1)	15 (20.0)
Job status				
Unemployed – housekeeper	89	20 (23.8)	7 (18.4)	13 (28.9)
Stable job status (Governmental employed/self-employed)	303	129 (42.6)	114 (43.9)	12 (30.8)
Unstable job status (Labourer/others)	98	29 (30.2)	27 (30.3)	1 (16.7)
Students	141	44 (31.9)^f^	34 (37.4)^f^	10 (21.3)
Marital status				
Currently unmarried (Single and previously married)	447	158 (36.1)	135 (39.1)	21 (23.6)
Currently married	177	62 (35.2)	46 (36.5)	14 (29.2)
Age at first sex				
≤18 years	170	60 (35.5)	48 (35.6)	11 (34.4)
>18 years	241	90 (37.7)	73 (44.5)	17 (22.7)
Experienced sex under the influence of alcohol or high on drugs
No/not remember	369	148 (39.7)	121 (42.3)	24 (28.9)
Yes	246	74 (29.9)^f^	61 (32.1)^f^	12 (21.8)
Condom knowledge				
Condom-related Knowledge question 1^b^
No	59	15 (25.9)	11 (23.4)	3 (30.0)
Yes	570	204 (36.4)	168 (39.3)^f^	33 (25.6)
Condom-related knowledge question 2^c^
No	159	38 (24.1)	31 (28.7)	6 (12.5)
Yes	469	181 (39.4)^f^	149 (40.7)^f^	29 (32.2)^f^
Condom knowledge scale^d^
No	183	48 (26.4)	39 (30.2)	7 (14.0)
Yes	411	168 (38.9)^f^	138 (40.5)^f^	28 (31.8)^f^
Knowledge on sexual route of HIV transmission				
No	154	39 (25.5)	34 (27.4)	3 (11.5)
Yes	474	180 (38.7)^f^	146 (41.7)^f^	32 (28.6)^e^

^a^ The prevalence of condom use overall and stratified by gender across the covariates is presented here; ^b^ Condomless sex, with an HIV-positive partner can transmit the HIV infection; ^c^ HIV can be prevented by properly using condom during sex; ^d^ Aggregated scale of the two previous questions; ^e^*P* value < .10; ^f^*P* value < .05

### Condom Use Patterns in the Sample

Of the 633 participants in the study, 222 (35.1%) subjects reported using a condom at last sex. In those who did not use a condom, the main reasons were “I did not think it was needed” (41.5%), “condom was not accessible at that time” (22.1%) and “I do not like condoms” (21%); other reasons were “my sexual partner disagreed” (8.1%), “we used other methods rather than condom” (4%) and “condom is expensive” (3.2%). Condom use at last sex was significantly higher among participants who had a governmental job or those who were self-employed (42.6% vs. 23.8% for unemployed individuals), no experience of sex under the influence of substance (39.7% vs. 29.9%), and had better knowledge of condom efficacy (38.9 vs. 26.4%) and sexual route of HIV infection (38.7% vs. 25.5%) (Table 1).

### Condom Use by Gender

Men reported significantly higher condom use at last sex than women (38.5% vs. 25.7%). Among men, those who had governmental/self-employed jobs (43.9% vs. 18.4% for unemployed individuals), did not experience sex under the influence of substances (42.3% vs. 32.1%), had better overall knowledge of condoms and sexual route of HIV transmission (40.5% vs. 30.2%), and reported higher frequency of condom use at last sex. Among women, those who had better knowledge of condoms (31.8% vs. 14.0%) reported more condom use at last sex (Table 1).

### Regression Analysis

In univariate regression analysis, being men, older age, stable job status, sufficient condom-related knowledge and having knowledge on sexual route of HIV transmission were positively associated with condom use at last sex. Conversely, reported experience of sex under the influence of substances had a negative association with condom use at last sex (Table 2). In multivariable analysis, it was shown that participants with more stable job status were 86% more likely to use a condom at last sex than unemployed/housekeeper subjects (AOR: 1.86; 95% CI: 1.01, 3.43). Those who had a better knowledge of condom use were 57% more likely to use a condom at last sex (AOR: 1.57; 95% CI: 1.03, 2.37). Moreover, those with better knowledge of HIV transmission route were 83% more likely to report condom use at last sex (AOR: 1.83; 95% CI: 1.18, 2.85). Those participants who reported having sex while drunk or high were less likely to use condoms (AOR: 0.66; 95% CI: 0.45, 0.94) (Table 2).

**Table 2:** Assessing Demographic and Knowledge Factors Associated With Condom Use at Last Sex Among Iranian Young Adults Aged 19-29 Years Old

**Variables**	**Crude Analysis** **OR (95% CI)**	**Adjusted Analysis** **OR (95% CI)**
Age		
19-24	1	1
25-29	1.36 (0.98, 1.90)^a^	1.21 (0.83, 1.75)
Gender		
Women	1	1
Men	1.76 (1.15, 2.68)^b^	1.53 (0.95, 2.46)^a^
Educational attainment		
Illiterate	1	
Diploma and less	0.74 (0.32, 1.72)	
Undergraduate	0.68 (0.31, 1.47)	
Graduate	0.71 (0.33, 1.53)	
Job status		
Unemployed – housekeeper	1	1
Stable job status (Governmental employed/self-employed)	2.30 (1.31, 4.0)^b^	1.86 (1.01, 3.43)^b^
Unstable job status (Labourer/others)	1.32 (0.67, 2.60)	1.09 (0.52, 2.27)
University students	1.47 (0.80, .73)	1.31 (0.67, 2.55)
Marital status		
Currently unmarried (Single and previously married)	1	
Currently married	0.93 (0.64, 1.35)	
Age at first sex		
≤18	1	
>18	1.10 (0.73, 1.70)	
Experienced sex under the influence of substances		
No	1	1
Yes	0.65 (0.46, 0.92)^b^	0.66 (0.45, 0.94)^b^
Condom-related knowledge (condom knowledge scale)		
Insufficient knowledge	1	1
Sufficient knowledge	1.82 (1.24, 2.70)^b^	1.57 (1.03, 2.37)^b^
Knowledge on sexual route of HIV transmission		
No	1	1
Yes	1.91 (1.26, 2.90)^b^	1.83 (1.18, 2.85)^b^

Abbreviation: OR, odds ratio. ^a^ *P* value < .10; ^b^*P* value < .05

## Discussion

We found that only one out of every three young adults reported using condoms at last sex. Believing that condoms are unnecessary, unavailable during intercourse, and not pleasant to use were reported as the main reasons for not using condoms among participants.

Low rates of condom use have been reported in previous studies in similar settings. For example, a recent study of 1076 young adults in Shiraz found that almost half of single individuals reported premarital sex, of whom around 24% had never used condoms and only about 42% had used it inconsistently.^31^ Another study in 2012 in Tehran on 3128 sexually active young men and women reported that only one-third of them had used condoms at last sex.^7^ One notable variable that affected condom use at last sex was employment status; participants who had stable jobs were significantly more likely to use condoms compared to unemployed/housekeepers young people. This might indicate that individuals with higher levels of economic status may be able to afford condoms and negotiate safe sex more effectively.^32^ We could not find any association between education and condom use in our study. This can probably be explained by the fact that sex education is missing from the educational institutions in Iran. Indeed, existing sexual health education programs such as mandatory premarital sex education are only targeted at married couples and neglect young and single populations.^33^ Moreover, these programs would not often cover the sexual health needs of those who might have extra marital sex, perhaps due to cultural taboos and religious values in the country.^29,34^

We also found that women were less likely to have used condoms at last sex. Gender norms – predominantly favouring men in Iran – can have a negative influence on sexual health by causing an inability to negotiate condom use and other reproductive health hazards among women.^7^ Socio-economic disparities that are caused by gender differences can also create such failure in condom use among women.^35,36^ However, a recent study among adult people in Iran showed that women had a better knowledge about sexual and reproductive health (SRH) compared to men.^12^ These discrepancies might represent the gap between awareness and practice related to SRH among women. Hence, practice-based approaches like empowerment and self-efficacy training which help develop healthy interpersonal skills^37,38^ are needed particularly among young women.

Having better knowledge of condom effectiveness and sexual route of HIV infection were other associated factors to condom use among young adults in our study. In fact, more knowledgeable individuals were more likely to use condoms at last sex. However, only three-quarters of the study participants had sufficient knowledge about condom use which is unsatisfactory among this vulnerable group. Educational interventions particularly sexual health education have been shown to reduce risk behaviour and lower the rates of STIs among young populations^39,40^; although the country still lacks youth friendly sexual health education and advice services.^11,13^ Conservative authorities in the country believe that sexual health education may motivate young people to engage in sexual activities at a younger age; this unproven claim has been criticized by researchers who emphasise that young generations can easily get information on sexual issues. However, they usually find such information from unreliable sources and laden with misconceptions and stigmatised beliefs.^26,41^ For example, in a recent nationwide study among young adults aged 15-29 years old, more than two-thirds had a misconception about HIV transmission through insect bites and around half of these people thought that getting HIV is a punishment for bad behaviour and sins.^26^ Sexual health education on risky behaviour reduction among young adults is more likely to be effective if it occurs before they become sexually active. In fact, early-initiated education will help to establish healthy sexual behaviours and will help young people make informed choices before having sex.^38,42^

In the context of high-risk sexual behaviour, the results showed that those who had experienced sex under the influence of alcohol or drugs were less likely to use condom at last sex. This is consistent with evidence suggesting that sex under the influence of substance increases the risk for involvement in high-risk sexual behaviours such as condomless sex, inconsistent condom use, and multiple sexual partnerships.^43-45^ Simultaneity of these two risky behaviours among young adults is a serious concern since the estimated prevalence of this phenomenon in our study was considerably high. Given that both risky sexual behaviours and substance use have been on the rise among young adults during the last decades in Iran,^46-48^ our findings call for further research to shed more light on our understanding of sex under the influence of drugs among young people in Iran.

We would like to acknowledge the limitations of our study. Due to the cross-sectional nature of the study, we could not assess the determinants of condom use over time. Condom use was measured the last time participants had penetrative sex. Although this is a commonly used proxy measure of condom use in sexual behaviour studies since it is less likely to be subject to recall bias,^49^ the term “penetrative sex” might have been misinterpreted by participants and consequently affected the estimate of condom use in the study sample. Moreover, self-reported data on sensitive information such as sexual activities or high-risk behaviours are subject to social desirability bias. In fact, the tendency to hide such behaviours may lead to underreporting and could violate the accuracy of such information.^1^ We defined “Extramarital sex” as having sex outside the context of marriage regardless of one’s marital status, however the risk cannot be deemed to be the same for those engaging in extra-marital affairs vs. premarital sex without further investigations. We also did not differentiate between condom use practices within different types of sex (ie, oral, anal, or vaginal) or sexual partners (eg, close or loyal friends, paid or unpaid partners). Sexually-related behaviours are highly stigmatized behaviours in the context of Iran so that people may not comfortably answer to such questions; therefore, we restricted our questions to more general questions and tried to address the main concerns of the health authorities in the Ministry of Health.

## Conclusion

The present study adds to the existing narrow body of literature on the sexual health of young populations in Iran. In the youth population having extramarital sex, only one-third reported condom use at last sex which is low. While motivational educational interventions such as programs that encourage correct and consistent condom use and promote condom negotiation confidence and skills are helpful, strategies considering multi-sectoral approaches including individual-, community-, and structural-level interventions are essential to bring about behaviour change and greatest impact. Future research in the area of condom use in Iran should focus on youth-centered, gender-specific, and gender-sensitive interventions considering multiple strategies. In addition, novel strategies such as mass media communications and social media campaigns that target youths with different socio-economic contexts are other effective strategies that have not yet been used in Iran.

## Acknowledgements

This study was financially supported by UNICEF (Grant number: 26210.1). We thank the participants of the study for spending their valuable time to provide such valuable information. We have to also thank our colleagues and experts in Ministry of Health of Iran for providing very helpful insights and feedback that greatly assisted the study. Lastly, we are thankful to the interviewers for their efforts on data collection process. Mohammad Karamouzian is supported through the Vanier Canada Graduate Scholarship and Pierre Elliott Trudeau Foundation Doctoral Scholarship.

## Ethical issues

All information was anonymously obtained from participants, and they were fully assured that their personal information would remain confidential and data would be used for research purposes only. Informed verbal consent was obtained from all participants. The study was reviewed and approved by Iran’s MoHME, Tehran, Iran and the ethics research committee of Kerman University of Medical Sciences, Kerman, Iran.

## Competing interests

Authors declare that they have no competing interests.

## Authors’ contributions

Study design: HS, MS, SHH; Data collection: HS; Data analysis: SHH, MS; Data interpretation: SHH, AM, MS; Manuscript drafting: SHH, MS; Critical revision of the manuscript: MK, AM, AAH, HS, MS; Final approval of the revision to be published: All co-authors approved the content of this manuscript. Authors SHH and MS contributed equally to this manuscript.

## Authors’ affiliations

^1^HIV/STI Surveillance Research Center, and WHO Collaborating Center for HIV Surveillance, Institute for Futures Studies in Health, Kerman University of Medical Sciences, Kerman, Iran. ^2^The Kirby Institute, UNSW Sydney, Sydney, NSW, Australia. ^3^School of Population and Public Health, Faculty of Medicine, University of British Columbia, Vancouver, BC, Canada. ^4^Department of Epidemiology and Biostatistics, University of California San Francisco, San Francisco, CA, USA. ^5^Department of Epidemiology & Biostatistics, Schulich School of Medicine & Dentistry, The University of Western Ontario, London, ON, Canada.

## Key messages

**Implications for policy makers**

- Only one third of sexually active young adults reported condom use at last sex. This rate is low and needs to be addressed in future programming for sex education and counselling.
- Young women were found to be less likely to use a condom at last sex. Effective gender-specific interventions should be provided for young women.
- Young people who knew more about condoms were more likely to have used it at last sex. Youth-specific sexual health educational interventions are required to raise awareness about the importance of condom use among this population.
- Sex under the influence of alcohol or drugs was frequent and associated with reduced condom use among young adults. This calls for the development of strategies that will help improve young people’s awareness of the consequences of such risky practices.

**Implications for the public**

Given the low rate of condom use among young adults in Iran, educating them about the importance of having conversations about safe sex practices with their partners and potential consequences of condomless sex are essential. Young women who have a lower rate of condom use need to be equipped with safe sex negotiation skills.
